# Diagnostic accuracy of a three-gene *Mycobacterium tuberculosis* host response cartridge using fingerstick blood for childhood tuberculosis: a multicentre prospective study in low-income and middle-income countries

**DOI:** 10.1016/S1473-3099(23)00491-7

**Published:** 2024-02

**Authors:** Laura Olbrich, Valsan P Verghese, Zoe Franckling-Smith, Issa Sabi, Nyanda E Ntinginya, Alfred Mfinanga, Denise Banze, Sofia Viegas, Celso Khosa, Robina Semphere, Marriott Nliwasa, Timothy D McHugh, Leyla Larsson, Alia Razid, Rinn Song, Elizabeth L Corbett, Pamela Nabeta, Andre Trollip, Stephen M Graham, Michael Hoelscher, Christof Geldmacher, Heather J Zar, Joy Sarojini Michael, Norbert Heinrich, RaPaed-TB consortium, Cynthia Biddle Baard, Jacinta Diane Munro, Margaretha Prins, Nolufefe Benzi, Linda Claire Bateman, Ashleigh Ryan, Kutala Booi, Nezisa Paulo, Anthenette Heydenrych, Wonita Petersen, Raquel Brookes, Michele Mento, Chad Centner, Craig Dalgarno, Friedrich Rieß, Sarah Mutuku, Elmar Saathoff, Kathrin Held, Marilyn Mary Ninan, Anila Chacko, Ramya Kumari, R Dhanabhagyam, Nithya Muniswamy, Marc P Nicol, Bariki Mtafya, Harieth Mwambola, Christina Manyama, Hellen Mahiga, Emanuel Sichone, Lwitiho Sudi, Cremildo Maueia, Carla Madeira, Justina Cambuie, Jorge Ribeiro, Lingstone Chiume, Alice Mnyanga, Tionge Sikwese, Happy Masakasa, Diana Kachere, Masheck Kosaka, Stefan Niemann, Novel Chegou, Lyn Horn

**Affiliations:** a Division of Infectious Diseases and Tropical Medicine, LMU University Hospital, LMU Munich, Munich, Germany; b CIHLMU Center for International Health, LMU University Hospital, LMU Munich, Munich, Germany; c German Centre for Infection Research (DZIF), Partner Site Munich, Munich, Germany; d Fraunhofer Institute ITMP, Immunology, Infection and Pandemic Research, Munich, Germany; e Oxford Vaccine Group, Department of Paediatrics and the NIHR Oxford Biomedical Research Centre, University of Oxford, Oxford, UK; f Pediatric Infectious Diseases, Department of Pediatrics, Christian Medical College, Vellore, India; g Department of Paediatrics and Child Health, SA-MRC Unit on Child and Adolescent Health, University of Cape Town, Cape Town, South Africa; h Mbeya Medical Research Centre, National Institute for Medical Research, Mbeya, Tanzania; i Instituto Nacional de Saúde, Marracuene, Mozambique; j Helse Nord Tuberculosis Initiative, Department of Pathology, Kamuzu University of Health Sciences, Blantyre, Malawi; k Centre for Clinical Microbiology, University College London, London, UK; l Clinical Research Department, London School of Hygiene & Tropical Medicine, London, UK; m Foundation for Innovative New Diagnostics (FIND), Geneva, Switzerland; n Department of Paediatrics, University of Melbourne and Murdoch Children's Research Institute, Melbourne, VIC, Australia; o Unit Global Health, Helmholtz Zentrum München, German Research Center for Environmental Health (HMGU), Neuherberg, Germany; p Department of Clinical Microbiology, Christian Medical College, Vellore, India

## Abstract

**Background:**

Childhood tuberculosis remains a major cause of morbidity and mortality in part due to missed diagnosis. Diagnostic methods with enhanced sensitivity using easy-to-obtain specimens are needed. We aimed to assess the diagnostic accuracy of the Cepheid *Mycobacterium tuberculosis* Host Response prototype cartridge (MTB-HR), a candidate test measuring a three-gene transcriptomic signature from fingerstick blood, in children with presumptive tuberculosis disease.

**Methods:**

RaPaed-TB was a prospective diagnostic accuracy study conducted at four sites in African countries (Malawi, Mozambique, South Africa, and Tanzania) and one site in India. Children younger than 15 years with presumptive pulmonary or extrapulmonary tuberculosis were enrolled between Jan 21, 2019, and June 30, 2021. MTB-HR was performed at baseline and at 1 month in all children and was repeated at 3 months and 6 months in children on tuberculosis treatment. Accuracy was compared with tuberculosis status based on standardised microbiological, radiological, and clinical data.

**Findings:**

5313 potentially eligible children were screened, of whom 975 were eligible. 784 children had MTB-HR test results, of whom 639 had a diagnostic classification and were included in the analysis. MTB-HR differentiated children with culture-confirmed tuberculosis from those with unlikely tuberculosis with a sensitivity of 59·8% (95% CI 50·8–68·4). Using any microbiological confirmation (culture, Xpert MTB/RIF Ultra, or both), sensitivity was 41·6% (34·7–48·7), and using a composite clinical reference standard, sensitivity was 29·6% (25·4–34·2). Specificity for all three reference standards was 90·3% (95% CI 85·5–94·0). Performance was similar in different age groups and by malnutrition status. Among children living with HIV, accuracy against the strict reference standard tended to be lower (sensitivity 50·0%, 15·7–84·3) compared with those without HIV (61·0%, 51·6–69·9), although the difference did not reach statistical significance. Combining baseline MTB-HR result with one Ultra result identified 71·2% of children with microbiologically confirmed tuberculosis.

**Interpretation:**

MTB-HR showed promising diagnostic accuracy for culture-confirmed tuberculosis in this large, geographically diverse, paediatric cohort and hard-to-diagnose subgroups.

**Funding:**

European and Developing Countries Clinical Trials Partnership, UK Medical Research Council, Swedish International Development Cooperation Agency, Bundesministerium für Bildung und Forschung; German Center for Infection Research (DZIF).

## Introduction

Approximately 12% of people with tuberculosis worldwide are children younger than 15 years, with 1·2 million cases of tuberculosis disease among children each year.^1^ An estimated 240 000 children are projected to die annually, accounting for 16% of all tuberculosis-related deaths, with 80% of these occurring in those younger than 2 years.^1^, ^2^ The burden of disease and mortality is greatest in low-income and middle-income countries where challenges and delays, particularly in tuberculosis case detection, contribute to poor outcomes.

Tuberculosis diagnosis in children remains difficult. Signs and symptoms are non-specific and confirmation of disease by a microbiological reference standard is infrequent.^3^ The reported sensitivity of direct pathogen detection ranges from 2% to 40%, even in high-resource settings.^4^, ^5^ WHO has approved both tuberculosis culture and molecular rapid diagnostic tests, such as Xpert MTB/RIF Ultra (Cepheid, Sunnyvale, CA, USA; hereafter referred to as Ultra), but these are suboptimally sensitive because paucibacillary and extrapulmonary presentations are common.^3^, ^5^, ^6^, ^7^ Collection methods of recommended respiratory specimens are invasive and might be demanding in terms of infrastructure and skilled staff.^6^, ^8^ This deficiency of diagnostic reference standards renders paediatric diagnostic validation studies particularly challenging.

**Research in context**

**Evidence before this study**

Novel diagnostic tests using non-sputum specimens for rapid and accurate tuberculosis diagnosis are urgently needed, especially for children. Host blood transcriptomic signatures can distinguish tuberculosis from other diseases and latent tuberculosis infection by using blood as an easy-to-collect, non-respiratory specimen type. Based on a multicohort analysis from 14 published datasets with more than 2500 samples originating from ten countries, Sweeney and colleagues derived a three-gene mRNA-signature, which was translated into the *Mycobacterium tuberculosis* Host Response prototype cartridge (MTB-HR) to be performed on the GeneXpert platform. The first prospective evaluation of MTB-HR in an adult cohort (75 patients with tuberculosis and 120 patients with other respiratory diseases) indicated a promising diagnostic accuracy, with an area under the receiver operating characteristic curve of 0·88 (95% CI 0·83–0·94), 80% sensitivity (95% CI 76–85), and 94% specificity (95% CI 91–96) compared with a composite microbiological reference standard.

We searched PubMed for articles published in any language between database inception in 1996 and Feb 27, 2023, using the search terms “TB”, “Transcriptomics”, “Transcriptome”, “Host blood transcriptional signatures”, “Host response cartridge”, and “Accuracy”, in various combinations. From this search, as well as cross-referencing within the articles, we were able to include studies that assessed the performance of a three-gene (*GBP5, DUSP3*, and *KLF2*) signature in diagnosing tuberculosis disease compared with uninfected controls, latent tuberculosis, and other respiratory diseases, using a microbiological reference standard of a positive *M tuberculosis* culture, positive Xpert MTB/RIF Ultra assay, or both. These studies were largely conducted in adults and only a few were prospective. A repeat search on the same date with the added search terms “Child”, “Children”, “Paediatric”, or “Pediatric” yielded no results.

**Added value of this study**

To the best of our knowledge, this is the first study to assess the performance of the three-gene signature using the MTB-HR in a prospectively recruited cohort of children with presumptive pulmonary or extrapulmonary tuberculosis. Applying the previously published cutoff (TB-score of 1·5), MTB-HR was reliably able to differentiate children with culture-confirmed tuberculosis from those unlikely to have tuberculosis, both in the overall cohort and in key subgroups of interest, including infants younger than 1 year, children living with HIV, children with severe acute malnutrition, or children with extrapulmonary tuberculosis manifestations. Accuracy was lower when using a composite microbiological reference standard (ie, culture positive, Ultra positive, or both) or a composite clinical reference standard, and there was a positive association between strength of microbiological detection and MTB-HR accuracy. Our results suggest a potential diagnostic value by combining one Ultra on a respiratory sample with one MTB-HR. Additionally, the MTB-HR has potential value in monitoring treatment response, addressing an important gap among available paediatric tuberculosis diagnostic tests. Lastly, we explored the use of optimised cutoffs, suggesting a benefit from identifying a more suitable child-specific MTB-HR cutoff.

**Implications of all the available evidence**

Given the widespread availability of the GeneXpert platform, the short time to result, ease of using capillary blood, and the reported diagnostic yield, MTB-HR is a promising tool to substantially improve tuberculosis diagnosis for children, which is currently the main obstacle to reducing child tuberculosis mortality.

To address the need for better diagnostics, non-sputum-based assays with enhanced sensitivity are needed, especially for key subgroups, including infants younger than 1 year, children with severe acute malnutrition, children living with HIV, and children in resource-limited settings. In these subgroups, conventional diagnosis is particularly challenging, as highlighted by WHO's high-priority target product profiles for new tuberculosis diagnostics, which do not specify a minimum sensitivity for a paediatric tuberculosis diagnostic test, but an optimal sensitivity of at least 66%.^9^ However, currently available WHO-endorsed tests using child-friendly samples do not meet this target, with sensitivities of 56% reported for Ultra on stool and 44% on nasopharyngeal aspirate compared with culture, and even candidate tests such as the urine FujiLAM (Fujifilm, Tokyo, Japan) showing sensitivities of 42–63% compared with composite microbiological reference standards.^10^, ^11^

Host gene transcriptional signatures can distinguish between tuberculosis disease and other illnesses and are measured in blood, a sample easier to obtain than sputum in children.^12^, ^13^, ^14^ The Cepheid *Mycobacterium tuberculosis* Host Response prototype cartridge (MTB-HR) is a GeneXpert-based RT-PCR test assessing relative messenger RNA levels from fingerstick whole-blood samples.^14^, ^15^ It distinguishes tuberculosis from other diseases based on the expression of *GBP5* and *DUSP3* (upregulated in tuberculosis), and *KLF2* (downregulated in tuberculosis). The platform automatically calculates an MTB-HR TB-score based on cycle threshold (Ct) values. A previously published cutoff (1·5) was derived by applying Youden's index to determine optimal sensitivity and specificity to meet the WHO threshold for a triage test (90% sensitivity and 70% specificity).^15^ The first prospective evaluation in an adult cohort suggested an area under the receiver operating characteristic (ROC) curve (AUC) of 0·88 (95% CI 0·83–0·94) against a reference standard of Ultra and culture, with 80% sensitivity (95% CI 76–85) and 94% specificity (95% CI 91–96), and an AUC of 0·94 (95% CI 0·91–0·97) against Ultra alone.^15^ In this study, we present the first diagnostic accuracy assessment of MTB-HR in a large, prospective cohort of children with presumptive tuberculosis disease recruited in five low-income and middle-income countries.

## Methods

### Study design and population

RaPaed-TB was a prospective, multicentre diagnostic accuracy study aiming to evaluate novel diagnostic tests and testing approaches for tuberculosis in children with presumptive tuberculosis disease. The study protocol and methods are published elsewhere.^16^ The study was performed in accordance with the Declaration of Helsinki.^17^ The protocol and informed consent documents were approved by institutional review boards at all sites and the coordinating site (Division of Infectious Diseases and Tropical Medicine, Ludwig Maximilian University of Munich, Munich, Germany). The study is registered on ClinicalTrials.gov, NCT03734172.

Briefly, children younger than 15 years were recruited in participating health facilities between Jan 21, 2019, and June 30, 2021. These centres included three tertiary hospitals in South Africa, Malawi, and India, and two urban health facilities in Tanzania and Mozambique. Children were recruited in both inpatient (South Africa, Malawi, India, and Mozambique) and outpatient (Malawi, Tanzania, and Mozambique) settings. The inclusion and exclusion criteria are listed in the appendix (p 2); children were eligible for recruitment if they had at least one of: microbiological confirmation of tuberculosis disease, signs or symptoms of pulmonary tuberculosis disease, or signs or symptoms of extrapulmonary tuberculosis disease. Children were excluded if they weighed less than 2 kg, had received three or more doses of tuberculosis medication, or if study procedures were considered an undue risk to the child.^16^ Parents or guardians provided written informed consent, and participants provided written assent when they were older than the locally specified age for doing so.

### Procedures

After extensive training of all site staff, a standardised clinical, radiological, and laboratory workup was performed; monitoring and retraining, if necessary, was performed every 6 months.^16^ For the two respiratory specimens (spontaneous or induced sputum at African sites, or gastric lavage at the Indian site) obtained from each participant, solid and liquid media cultures (Mycobacteria Growth Indicator Tube; Becton Dickinson, Sparks, NJ, USA) were inoculated and one Ultra was performed. Children younger than 5 years underwent nasopharyngeal aspirate for a second Ultra. Children were followed up at 1 month and 3 months, and at 6 months if on tuberculosis treatment or still unwell at 3 months.

MTB-HR was done at baseline and repeated at 1 month, and additionally at 3 months and 6 months if on tuberculosis treatment. Fingerstick blood samples of 100 μL were taken, instilled into the MTB-HR cartridge within 15 min, and loaded onto the GeneXpert platform within 1 h of blood draw. Capillary samples taken before the MTB-HR cartridge became available on site were transferred into PAXgene buffer (Qiagen, Hilden, Germany) within 15 min of collection and stored at –80°C. Ct values for individual genes (*DUSP, GBP5*, and *KLF2*) were obtained. In case of an invalid result, the test was repeated on the same day or the next day, if possible. Laboratory staff were masked to the clinical findings and clinicians were masked to MTB-HR results. An MTB-HR TB-score was provided by the GeneXpert instrument ([Ct *GBP5* + Ct *DUSP3*]/2 – Ct *KLF2*). A tentative previously published cutoff was used, with less than 1·5 representing a positive result for tuberculosis disease.^15^ To account for variations due to storage, a correction factor based on the strict reference standard was derived by determining the difference of mean MTB-HR TB-score between samples stored in PAXgene buffer (hereafter referred to as biobanked samples) and prospectively tested samples, and then applied to biobanked samples.

Diagnostic classifications were adapted from the US National Institutes of Health consensus statement for paediatric tuberculosis diagnostic evaluation studies.^2^, ^16^ Clinical case definitions were used to derive reference standards for diagnostic accuracy estimation, which are described in detail in the appendix (p 3). Briefly, we defined a strict reference standard (SRS) including only children with culture or cultures positive for *M tuberculosis* as confirmed tuberculosis, compared with children with unlikely tuberculosis. Additionally, we evaluated diagnostic accuracy using a composite microbiological reference standard (MRS), including children with a positive Ultra, culture, or both. Lastly, a composite clinical reference standard (CRS) was defined, whereby children classified as confirmed tuberculosis or unconfirmed tuberculosis were regarded as positive.

### Statistical analysis

Diagnostic accuracy was tested using the SRS, MRS, and CRS. Differences in MTB-HR readout (gene transcripts and MTB-HR TB-score) between diagnostic categories were assessed by Mann-Whitney *U* tests. AUC was used to determine the accuracy of the MTB-HR TB-score and 95% CIs were calculated. Between-group AUCs were compared using the DeLong method. Sensitivity and specificity were calculated using the previously proposed cutoff of 1·5 and shown together with AUC for predefined subgroups of interest.^15^ To explore different cutoffs, we calculated the maximum Youden's index of the AUC and generated cutoffs with fixed sensitivity at 65% and at 90%.^18^ When plotting MTB-HR TB-scores over time, a one-way repeated measures ANOVA with pair-wise paired *t* tests was used to evaluate the difference between timepoints (appendix p 4). To account for heterogeneity between sites, a random-effects meta-analysis was conducted. Finally, the incremental yield of combining MTB-HR and one Ultra on respiratory samples was computed, including all microbiologically confirmed (positive culture, Ultra, or both) as the overall population.

Data were cleaned and analysed using Stata v16.1 and R v4.1.3. ROC curves and DeLong tests were computed using the pROC package and the roc.test function; random-effect meta-analysis used the mada package and the reitsma function.

### Role of the funding source

The funders of the study (EDCTP2 and DZIF) had no role in study design, data collection, data analysis, data interpretation, or writing of this report. Cepheid, which supplied testing kits at no cost, was given the opportunity to comment on the manuscript before submission.

## Results

**Figure 1:**
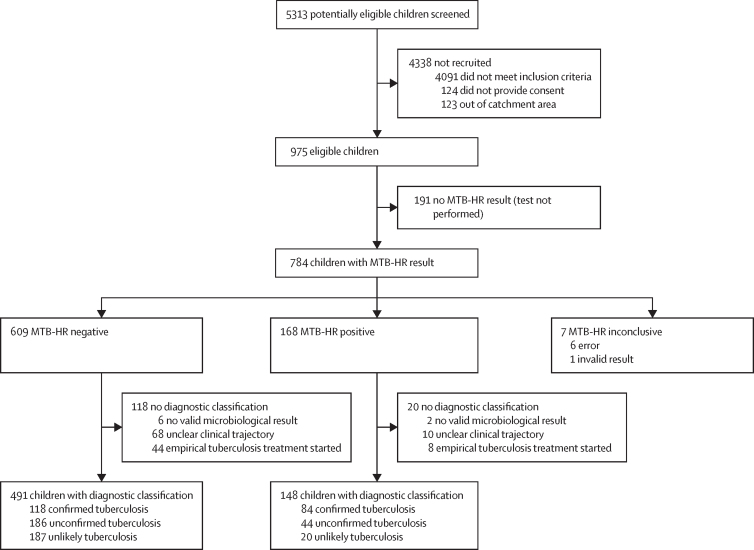
Flow diagram of participants in RaPaed-TB Definitions of clinical case classification are presented in the appendix (p 3). All children with defined clinical case definition were included in the analysis. Of those excluded, most had an unclear clinical trajectory (ie, were lost to follow-up). MTB-HR=Cepheid *Mycobacterium tuberculosis* Host Response prototype cartridge.

5313 potentially eligible children were screened, of whom 975 children were eligible and provided consent. Of these, 191 did not have an MTB-HR test result available at enrolment, most frequently due to a lost sample shipment from the Malawian site (n=116). Of 784 MTB-HR tests, 609 (78%) were negative, 168 (21%) were positive, and seven (1%) were inconclusive. 138 children with an MTB-HR result had no diagnostic classification. 202 (32%) of 639 children included in the analyses had confirmed tuberculosis, 230 (36%) had unconfirmed tuberculosis, and 207 (32%) were classified as unlikely tuberculosis (figure 1).

Among the study population, median age was 5·4 years (IQR 1·8–9·2) across categories and was higher in children with confirmed tuberculosis (6·5 years, 1·8–11·8) than in children with unconfirmed tuberculosis (4·4 years, 1·7–8·2) or unlikely tuberculosis (5·5 years, 2·3–7·8). Of all children, 89 (14%) were living with HIV, 71 (11%) had severe acute malnutrition, and 13 (20%) were living with HIV and had severe acute malnutrition (table 1).

**Table 1:** Demographic and clinical characteristics

		**Confirmed tuberculosis (n=202)**	**Unconfirmed tuberculosis (n=230)**	**Unlikely tuberculosis (n=207)**	**All (n=639)**
Site					
	South Africa	69 (34%)	78 (34%)	29 (14%)	176 (28%)
	Tanzania	43 (21%)	57 (25%)	78 (38%)	178 (28%)
	Mozambique	20 (10%)	72 (31%)	59 (29%)	151 (24%)
	Malawi	11 (5%)	11 (5%)	28 (14%)	50 (8%)
	India	59 (29%)	12 (5%)	13 (6%)	84 (13%)
Sex					
	Female	96 (48%)	101 (44%)	109 (53%)	306 (48%)
	Male	106 (52%)	129 (56%)	98 (47%)	333 (52%)
Age, years		6·5 (1·8–11·8)	4·4 (1·7–8·2)	5·5 (2·3–7·8)	5·4 (1·8–9·2)
	<1	32 (16%)	32 (14%)	18 (9%)	82 (13%)
	1–4	23 (11%)	38 (17%)	29 (14%)	90 (14%)
	5–9	32 (16%)	51 (22%)	42 (20%)	125 (20%)
	10–14	43 (21%)	73 (32%)	86 (42%)	202 (32%)
Comorbidities					
	Severe acute malnutrition	31/195 (16%)	23/228 (10%)	17/205 (8%)	71/628 (11%)
	HIV	16/202 (8%)	55/230 (24%)	18/207 (9%)	89/639 (14%)
Tuberculosis laboratory findings					
	Culture positive only	31 (15%)	..	..	31 (15%)
	Ultra positive only	75 (37%)	..	..	75 (37%)
	Culture and Ultra positive	96 (48%)	..	..	96 (48%)
Tuberculosis disease					
	Pulmonary tuberculosis only	111/196 (57%)	174/222 (78%)	..	285/418 (68%)
	Extrapulmonary tuberculosis only	39/196 (20%)	20/222 (9%)	..	59/418 (14%)
	Pulmonary and extrapulmonary tuberculosis	46/196 (23%)	28/222 (13%)	..	74/418 (18%)
Tuberculosis-related clinical findings					
	Tuberculin skin test positive	113/182 (63%)	105/199 (53%)	81/192 (42%)	299/573 (52%)
	Non-severe tuberculosis disease*	41/196 (21%)	85/222 (38%)	..	126/418 (30%)
	Hospitalised at enrolment	105/202 (52%)	76/230 (33%)	44/207 (21%)	225/639 (35%)
	Chest x-ray findings consistent with tuberculosis†	94/202 (47%)	73/230 (32%)	29/207 (14%)	196/639 (31%)

Data are n (%), median (IQR), or n/N (%). Stratification of clinical and demographic characteristics was done by clinical case definition. Ultra=Xpert MTB/RIF Ultra diagnostic test.

* According to WHO definition,^18^ outlined in the appendix (p 3).

† According to masked expert reading.

**Figure 2:**
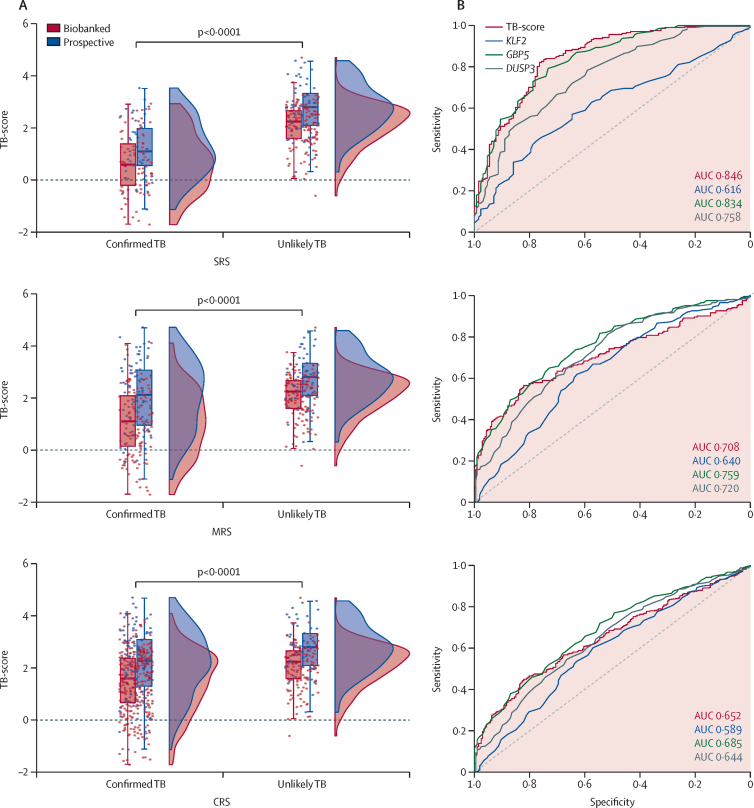
MTB-HR TB-score distribution of participants classified as having confirmed tuberculosis or unlikely tuberculosis by SRS, MRS, or CRS (A) Raincloud plots show the distribution of the TB-score for the SRS (top panel), MRS (middle panel), and CRS (bottom panel). The bars indicate the 10–90% percentile, and the box indicates the median and IQR. The points are individual data points and show the spread. The data are displayed stratified by whether the sample was prospective or biobanked (before recentring), but the statistical test was conducted on the entire sample, comparing confirmed tuberculosis with unlikely tuberculosis. Data were analysed using Mann-Whitney *U* tests. (B) AUC for the MTB-HR TB-score of all participants and gene transcripts for the SRS (top panel), MRS (middle panel), and CRS (bottom panel). For the SRS, DeLong tests between TB-score and gene transcripts were: *KLF2* p<0·0001, *DUSP3* p=0·0018, and *GBP5* p=0·53. For the MRS, DeLong tests between TB-score and gene transcripts were: *KLF2* p=0·094, *DUSP3* p=0·69, and *GBP5* p=0·018. For the CRS, DeLong tests between TB-score and gene transcripts were: *KLF2* p=0·067, *DUSP3* p=0·76, and *GBP5* p=0·12. AUC=area under the receiver operating characteristic curve. CRS=clinical reference standard. MRS=microbiological reference standard. MTB-HR=Cepheid *Mycobacterium tuberculosis* Host Response prototype cartridge. SRS=strict reference standard. TB=tuberculosis.

MTB-HR performance was calculated using three distinct reference standards: SRS, MRS, and CRS (appendix p 3). Median TB-scores differed between MTB-HR conducted on prospective (fresh) versus biobanked (stored) samples, with prospective samples showing overall higher median scores and Ct values for *DUSP3, GBP5*, and *KLF2* (figure 2). Recentred scores for biobanked samples were used for the rest of the analysis by applying the derived correction factor (0·533). AUCs and median Ct values for all three genes stratified by prospective versus biobanked samples and by reference standards are presented in the appendix (pp 5–6).

Across the cohort, using SRS (n=334), the MTB-HR TB-score discriminated between children with culture-confirmed tuberculosis (median TB-score 1·1, IQR 0·3–1·9) and children with unlikely tuberculosis (2·8, 2·1–3·2) at an AUC of 0·85 (95% CI 0·80–0·89; figure 2A) with a sensitivity of 59·8% (95% CI 50·8–68·4). Using MRS (n=409), the AUC was 0·71 (0·66–0·76) and sensitivity was 41·6% (34·7–48·7). Using CRS (n=639), the AUC was 0·65 (0·61–0·69) and sensitivity was 29·6% (25·4–34·2; figure 2B). Using all three reference standards, specificity was 90·3% (95% CI 85·5–94·0)

**Figure 3:**
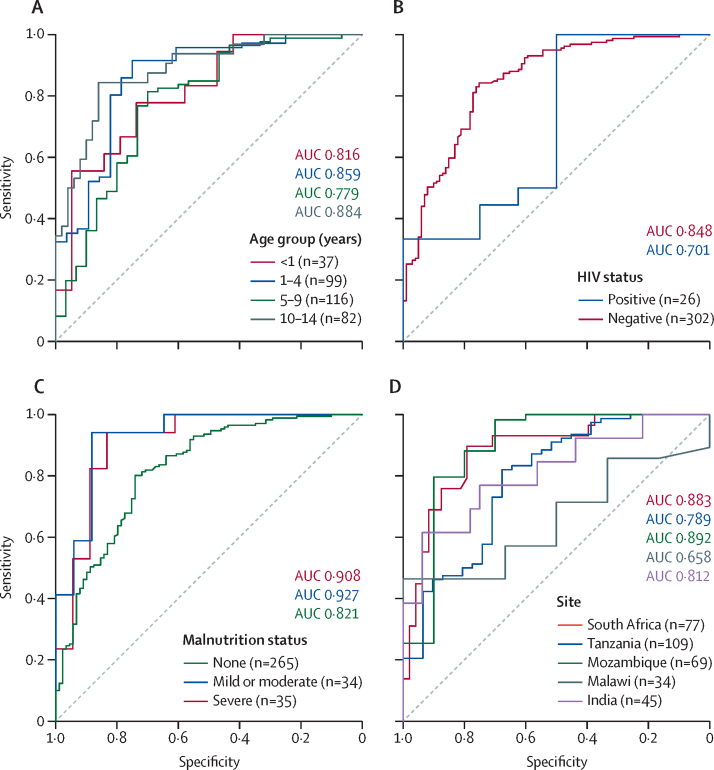
MTB-HR TB-score to estimate diagnostic accuracy of MTB-HR in subgroups of interest according to the strict reference standard (A) Diagnostic accuracy by age group (n=334). DeLong test between age <1 year and other age groups: 1–5 years p=0·60, 5–10 years p=0·68, >10 years p=0·63. (B) Diagnostic accuracy by HIV status (n=328). DeLong test between HIV negative and HIV positive: p=0·31. (C) Diagnostic accuracy by malnutrition status (n=334). DeLong test between no malnutrition and other malnutrition statuses: severe p=0·15, mild or moderate p=0·052. (D) Diagnostic accuracy by site (n=334). DeLong test between South Africa and other sites: Tanzania p=0·14, Mozambique p=0·92, Malawi p=0·047, India p=0·42. AUC=area under the receiver operating characteristic curve. MTB-HR=Cepheid *Mycobacterium tuberculosis* Host Response prototype cartridge.

Diagnostic accuracy of MTB-HR TB-score in key subgroups was assessed applying the SRS (figure 3, table 2; appendix pp 8–9), the MRS (appendix pp 8–10), and the CRS (appendix pp 8, 10). Performance differed slightly between subgroups without reaching statistical significance for any of the reference standards, including between age groups (DeLong p=0·055), HIV status (DeLong p=0·31), and malnutrition status (DeLong p=0·052)**.** Stratification by country suggested similar results for most sites except Malawi, which contributed only three culture-confirmed patients. The sensitivity analysis using random-effects meta-analysis for site heterogeneity resulted in a pooled sensitivity and specificity of 57·9% (95% CI 42·7–71·7) and 89·1% (95% CI 83·2–93·0), respectively, which was similar to the fixed-effects analysis, with a sensitivity and specificity of 59·8% (50·8–68·4) and 90·3% (85·5–94·0), respectively (appendix p 11).

**Table 2:** Diagnostic accuracy of MTB-HR at a cutoff of 1·5 against the strict reference standard using culture for different subgroups

		**Samples, n**					**Sensitivity, % (95% CI)**	**Specificity, % (95% CI)**
		All	True positive	False positive	False negative	True negative		
Overall		334	76	20	51	187	59·8% (50·8–68·4)	90·3% (85·5–94·0)
Age, years								
	<1	37	8	1	11	17	42·1% (20·3–66·5)	94·4% (72·7–99·9)
	1–4	99	19	6	9	65	67·9% (47·6–84·1)	91·5% (82·5–96·8)
	5–9	116	14	9	16	77	46·7% (28·3–65·7)	89·5% (81·1–95·1)
	10–14	82	35	4	15	28	70·0% (55·4–82·1)	87·5% (71·0–96·5)
HIV status								
	Negative*	302	72	17	46	167	61·0% (51·6–69·9)	90·8% (85·6–94·5)
	Positive	26	4	2	4	16	50·0% (15·7–84·3)	88·9% (65·3–98·6)
Malnutrition†								
	None	265	51	18	41	155	55·4% (44·7–65·8)	89·6% (84·1–93·7)
	Mild or moderate	34	13	1	4	16	76·5% (50·1–93·2)	94·1% (71·3–99·9)
	Severe	35	12	1	6	16	66·7% (41·0–86·7)	94·1% (71·3–99·9)
Site								
	South Africa	77	35	3	13	26	72·9% (58·2–84·7)	89·7% (72·6–97·8)
	Tanzania	109	15	6	16	72	48·4% (30·2–66·9)	92·3% (84·0–97·1)
	Mozambique	69	7	5	3	54	70·0% (34·8–93·3)	91·5% (81·3–97·2)
	Malawi	34	1	4	5	24	16·7% (0·4–64·1)	85·7% (67·3–96·0)
	India	45	18	2	14	11	56·3% (37·7–73·6)	84·6% (54·6–98·1)
Tuberculosis severity‡								
	Non severe§	21	9	..	12	..	42·9% (21·8–66·0)	..
	Severe¶	102	66	..	36	..	64·7% (54·6–73·9)	..
Tuberculosis location‡								
	Pulmonary tuberculosis	69	39	..	30	..	56·5% (44·0–68·4)	..
	Extrapulmonary tuberculosis	18	9	..	9	..	50·0% (26·0–74·0)	..
	Pulmonary and extrapulmonary tuberculosis	36	27	..	9	..	75·0% (57·8–87·9)	..
	Lymph node tuberculosis	20	14	..	6	..	70·0% (45·7–88·1)	..
	Tuberculosis meningitis	12	8	..	4	..	66·7% (34·9–90·1)	..

Totals might differ due to missing data. MTB-HR=Cepheid *Mycobacterium tuberculosis* Host Response prototype cartridge.

* Includes children who are HIV-exposed but not positive.

† According to WHO child growth standards.^19^

‡ Tuberculosis disease severity and location imply that the child already has tuberculosis; therefore, calculating a specificity was not possible.

§ According to WHO definition,^18^ outlined in the appendix (p 3).

¶ Children not meeting non-severe tuberculosis disease definition.

MTB-HR TB-score performance was stratified by disease severity, as per WHO definition,^18^ and organ manifestation of tuberculosis disease in children with culture-confirmed tuberculosis. We found a higher sensitivity in children with severe (64·7%, 95% CI 54·6–73·9) versus non-severe tuberculosis disease (42·9%, 21·8–66·0), and for disseminated disease—ie, both pulmonary and extrapulmonary tuberculosis (75·0%, 57·8–87·9) compared with only pulmonary (56·5%, 44·0–68·4) or only extrapulmonary disease (50·0%, 26·0–74·0).

MTB-HR TB-score was stratified by means of microbiological detection and strength of signal (appendix p 11). MTB-HR TB-scores did not change substantially between Mycobacteria Growth Indicator Tube time-to-positivity tertiles, but Ultra readouts and median MTB-HR TB-scores in children showed consistency, with very low or trace Ultra results being above the proposed cutoff of 1·5 (ie, the majority would have had a negative MTB-HR result).

We explored other cutoffs based on the MTB-HR results and diagnostic classifications. Based on the MTB-HR results in the SRS, the Youden's index proposed a cutoff at 1·99 with a sensitivity of 82·1% and a specificity of 76·4%. Setting the sensitivity at 65% resulted in a cutoff at 1·62 with a specificity of 87·9%, and setting the sensitivity at 90% resulted in a cutoff at 2·76 with a specificity of 51·2%.

To inform potential sampling and testing strategies, we assessed the incremental yield of MTB-HR and one Ultra on respiratory samples. Children were included if confirmed by Ultra or culture (or both) from sputum, gastric lavage, or nasopharyngeal aspirate. In older children aged 5–14 years, 50 (46%) of 109 were identified by a positive MTB-HR, with an additional 20 (18%) testing positive on sputum or gastric lavage Ultra. Tuberculosis culture identified five (5%) additional children (appendix p 12). Among young children (<5 years), 19 (32%) of 60 tested positive by MTB-HR, with an additional seven (12%) identified by one positive Ultra on nasopharyngeal aspirate, and an additional 20 (33%) by a positive Ultra on a respiratory sample. Thus, if MTB-HR were combined with one Ultra on sputum or gastric lavage, 43 (71%) of 60 children would have been identified, whereas adding a subsequent nasopharyngeal aspirate Ultra would have identified three (5%) additional children. The additional yield of a tuberculosis culture in addition to Ultra was low (one child, 2%; appendix p 12).

Lastly, longitudinal MTB-HR TB-scores were visualised for children with culture-confirmed tuberculosis (n=127) and children with unlikely tuberculosis (n=207; appendix p 12). MTB-HR TB-score increased in children treated for tuberculosis from baseline (median 1·1, IQR 0·3–1·9) to 1 month (1·9, 1·3–2·6), 3 months (2·4, 1·7–2·9), and 6 months (3·0, 2·5–3·3; ANOVA p<0·0001) but did not show significant changes over time in children with unlikely tuberculosis (baseline median TB-score: 2·8, 2·1–3·2; 1 month: 2·9, 2·4–3·2; 3 months: 3·0, 2·6–3·23; ANOVA p=0·079). Pairwise paired *t* tests showed significant differences in MTB-HR T-score among children who were culture positive between each visit (p≤0·05) and no significant differences between the visits of those who were culture negative. Children were then grouped by clinical characteristics and time to MTB-HR TB-score of more than 1·5 was calculated; most children younger than 1 year and with non-severe tuberculosis changed from a positive to a negative MTB-HR result within 1 month (appendix p 13).

## Discussion

Evidence presented in this study on the first prospective evaluation of the MTB-HR cartridge in children using the proposed TB-score of 1·5 suggests an AUC of 0·85, which is similar to that previously reported in adults (AUCs of 0·88 and 0·89).^15^, ^20^ Additionally, despite small differences, diagnostic accuracy was similar across age categories and in children with malnutrition, HIV, and extrapulmonary tuberculosis.

Currently available diagnostic approaches in children are either less sensitive (bacteriological) or less specific (clinical or radiological) than in adults.^3^, ^5^ This is underlined by the fact that WHO target product profiles for a paediatric test do not define a minimal sensitivity, but an optimal one of at least 66%.^9^ In our study, MTB-HR either meets or is close to this target in some subgroups, underlining the potential of rapid diagnosis. This result holds promise for addressing the diagnostic gap in children investigated for tuberculosis.

Semi-automated testing platforms such as GeneXpert facilitate a rapid turnaround time of complex assays in settings with limited resources. Paediatric care is often highly specialised in settings with a high burden of tuberculosis and is therefore centralised at secondary or tertiary levels of health services, although most children present at primary health care.^2^ Xpert MTB/RIF has been shown to be an efficient first-line test for paediatric tuberculosis by a large study in India, with a median duration between specimen collection and reporting of results of 0 days and almost 90% treatment initiation.^21^ However, despite upward trends, countries have been slow to roll out and scale up implementation of automated testing platforms, mostly due to purchase and distribution costs.^22^, ^23^ Poor sensitisation and training of clinical and laboratory staff result in underutilisation and inadequate exploitation, highlighting the need to improve the overall cascade of paediatric tuberculosis care.^24^ Even if these platforms are available, specimen collection remains a major hurdle.^6^, ^8^

Fingerstick blood is an attractive specimen type for a paediatric tuberculosis test, being much simpler and quicker to obtain than traditional respiratory specimens. It is generally well accepted by children and health-care workers, even at the primary health care level.^25^ Our results suggest that more than two-thirds of children with confirmed tuberculosis can be identified by the combination of one MTB-HR with one respiratory specimen tested on Ultra. Considering the operational characteristics of the platform, the rapid sample-to-result turnaround time of less than 1 h, and the diagnostic performance using the current cutoff, MTB-HR might be best used as a diagnostic instead of as a triage test.

In 2022, WHO recommended treatment decision algorithms for childhood tuberculosis at low levels of health care, such as primary health care clinics, in countries with high tuberculosis burden, and a point-of-care test with a high specificity, such as MTB-HR, could improve the diagnostic accuracy for clinical diagnosis.^18^ Additionally, we found that diagnostic accuracy was generally similar across subgroups of interest and in those difficult to diagnose, such as children who are severely malnourished. Thus, MTB-HR might be valuable as a treatment decision and monitoring tool, because a positive MTB-HR might be indicative of a more severe form of tuberculosis and could potentially inform treatment duration.

The three-gene mRNA signature used by MTB-HR is derived from a multicohort analysis from 14 published datasets with more than 2500 (mostly adult) samples.^14^ Similarly, the calculated MTB-HR TB-score was proposed based on an adult cohort.^15^ Exploratory analyses in our study suggest that an adaptation of the cutoff might be beneficial to optimise diagnostic performance, warranting further analyses.

Diagnostic accuracy studies in diseases with imperfect and unreliable reference standards, such as paediatric tuberculosis, might be suboptimally accurate, especially when composite reference standards are applied.^26^ Thus, we conducted our analysis reflecting the different levels of certainty: culture confirmation, composite microbiological confirmation, and composite clinical reference standard, all of which have limitations. Although culture might have a lower and heterogeneous yield compared with other methods, such as clinical diagnosis, it is widely accepted to be rarely false positive and has its own place in rigorous diagnostic accuracy evaluations.^27^ Relying on culture alone might skew the results towards multibacillary disease, and although Ultra has a reported higher sensitivity, uncertainties around specificity remain. In an updated Cochrane review, Ultra specificity in children ranged from 94·1% to 98·0%, depending on specimen type.^10^ Translated to our study where we conducted a total of 1467 Ultras, between 29 and 87 would be false positive. Similarly, the use of a composite clinical reference standard has considerable limitations. Although it is important to estimate the accuracy of a novel test in the group of children in most need for a new diagnostic approach (ie, unconfirmed tuberculosis), ascertaining a tuberculosis diagnosis is per definition impossible by combining sets of existing imperfect tests.^26^ A study in Lima, Peru, followed up 35 children meeting the criteria for unconfirmed tuberculosis that were not treated for a median time of 16 months, with only two developing tuberculosis disease.^28^ It is therefore likely that a proportion of children defined as unconfirmed tuberculosis did not have tuberculosis in our study, offering one explanation for the inferior diagnostic performance found using the composite reference standards.

This study had several limitations. Because we performed a rigorous diagnostic study in a well defined population of children investigated for tuberculosis disease, large proportions had confirmed and unconfirmed tuberculosis. This over-representation of true tuberculosis cases might overestimate MTB-HR performance in the target population of children presenting at primary health care. Additionally, some subgroups were small, such as children living with HIV, limiting the interpretability of accuracy estimates. Furthermore, to stringently assign the clinical case definitions, several children were not included, mostly due to unclear clinical trajectory (ie, lost to follow-up or lack of treatment response). One site was also heavily under-represented (Malawi) due to a lost shipment. Because the MTB-HR only became available in late 2019, our study dataset is composed of samples analysed immediately after collection but also following biobanking. We observed that there was a difference in median scores between the stored and the fresh samples, probably due to RNA degradation in biobanked samples.^29^ However, by recentring the means of the biobanked samples to the prospective samples, we found a similar distribution of scores between the two groups and the calculated AUCs were almost identical. This batch effect difference between stored and fresh samples could have been suboptimal for analysis of diagnostic accuracy. Lastly, MTB-HR was conducted by trained study laboratory staff and not routine clinical staff.

Being the first study on the use of this assay in children, future studies are required to replicate our findings. The use of an age-adjusted or child-specific cutoff needs to be explored, which should be prospectively tested, as well as the optimal placement within the health-care cascade, including within recently recommended treatment decision algorithms published by WHO.^30^ Implementation studies will be needed to assess performance at different levels of the care cascade and to test performance by health-care workers rather than laboratory staff.

In summary, our study presents the first estimates on diagnostic accuracy of the MTB-HR on a fingerstick blood sample within a large, well defined, and geographically diverse paediatric cohort. Considering its operational characteristics, MTB-HR has the potential to provide an urgently needed new approach to improve tuberculosis diagnosis for children globally.

## Data sharing

Data collected for the study include individual participant data and a data dictionary defining each field in the set. These will be made available in the form of de-identified data upon reasonable request made to the corresponding author. Therefore, approval of a proposal by the RaPaed-TB consortium will need to be sought and a data access or sharing agreement, following the guidelines of the EDCTP and the EU General Data Protection Regulation, will need to be signed.

## Declaration of interests

All authors declare receiving grant funding for this work from the second European and Developing Countries Clinical Trials Partnership programme (EDCTP2), the German Center for Infection Research (DZIF), and Beckman Coulter to their respective institutions. Cepheid provided testing kits at no cost. ZF-S and HJZ declare funding from the SA-MRC Unit on Child and Adolescent Health to their institution. TDM received funding to his institution from Médicins Sans Frontières 2017–23, GOSH Charity Intramural COVID-19 Rapid Response Funding, Global Alliance Against Tuberculosis, and EU Innovative Medicines Initiative for other research activities. TDM receives personal payment in his function as Editor in Chief of *Annals of Clinical Microbiology and Antimicrobials*.

## References

[1] WHO (2022).

[2] WHO (2018).

[3] Thomas TA (2017). Tuberculosis in children. Pediatr Clin North Am.

[4] Tebruegge M, Ritz N, Curtis N, Shingadia D (2015). Diagnostic tests for childhood tuberculosis: past imperfect, present tense and future perfect?. Pediatr Infect Dis J.

[5] Detjen AK, DiNardo AR, Leyden J (2015). Xpert MTB/RIF assay for the diagnosis of pulmonary tuberculosis in children: a systematic review and meta-analysis. Lancet Respir Med.

[6] Zar HJ, Hanslo D, Apolles P, Swingler G, Hussey G (2005). Induced sputum versus gastric lavage for microbiological confirmation of pulmonary tuberculosis in infants and young children: a prospective study. Lancet.

[7] WHO (2013).

[8] Thomas TA, Heysell SK, Moodley P (2014). Intensified specimen collection to improve tuberculosis diagnosis in children from Rural South Africa, an observational study. BMC Infect Dis.

[9] WHO (2014).

[10] Kay AW, Ness T, Verkuijl SE (2022). Xpert MTB/RIF Ultra assay for tuberculosis disease and rifampicin resistance in children. Cochrane Database Syst Rev.

[11] Olbrich L, Khambati N, Bijker EM, Ruhwald M, Heinrich N, Song R (2022). FujiLAM for the diagnosis of childhood tuberculosis: a systematic review. BMJ Paediatr Open.

[12] Zak DE, Penn-Nicholson A, Scriba TJ (2016). A blood RNA signature for tuberculosis disease risk: a prospective cohort study. Lancet.

[13] Anderson ST, Kaforou M, Brent AJ (2014). Diagnosis of childhood tuberculosis and host RNA expression in Africa. N Engl J Med.

[14] Sweeney TE, Braviak L, Tato CM, Khatri P (2016). Genome-wide expression for diagnosis of pulmonary tuberculosis: a multicohort analysis. Lancet Respir Med.

[15] Sutherland JS, van der Spuy G, Gindeh A (2022). Diagnostic accuracy of the Cepheid 3-gene host response fingerstick blood test in a prospective, multi-site study: interim results. Clin Infect Dis.

[16] Olbrich L, Nliwasa M, Sabi I (2023). Rapid and accurate diagnosis of pediatric tuberculosis disease: a diagnostic accuracy study for pediatric tuberculosis. Pediatr Infect Dis J.

[17] World Medical Association (2013). World Medical Association Declaration of Helsinki: ethical principles for medical research involving human subjects. JAMA.

[18] WHO (2022).

[19] WHO (2009).

[20] Södersten E, Ongarello S, Mantsoki A (2021). Diagnostic accuracy study of a novel blood-based assay for identification of tuberculosis in people living with HIV. J Clin Microbiol.

[21] Kalra A, Parija D, Raizada N (2020). Upfront Xpert MTB/RIF for diagnosis of pediatric TB—does it work? Experience from India. PLoS One.

[22] Adam P, Pai M (2019). Implementation of Xpert MTB/RIF in high-burden countries: voices from the field matter. Public Health Action.

[23] Ponnudurai N, Denkinger CM, Van Gemert W, Pai M (2018). New TB tools need to be affordable in the private sector: the case study of Xpert MTB/RIF. J Epidemiol Glob Health.

[24] Cazabon D, Pande T, Kik S (2018). Market penetration of Xpert MTB/RIF in high tuberculosis burden countries: a trend analysis from 2014–2016. Gates Open Res.

[25] Sutcliffe CG, Palamountain KM, Maunga S (2018). The feasibility of fingerstick blood collection for point-of-care HIV-1 viral load monitoring in rural Zambia. Glob Health Innov.

[26] Dendukuri N, Schiller I, De Groot J (2018). Concerns about composite reference standards in diagnostic research. BMJ.

[27] DiNardo AR, Detjen A, Ustero P, Ngo K, Bacha J, Mandalakas AM (2016). Culture is an imperfect and heterogeneous reference standard in pediatric tuberculosis. Tuberculosis (Edinb).

[28] Wong M, Coit JM, Mendoza M (2021). Incident tuberculosis diagnoses in children at high risk for disease. Open Forum Infect Dis.

[29] Wilson C, Dias NW, Pancini S, Mercadante V, Biase FH (2022). Delayed processing of blood samples impairs the accuracy of mRNA-based biomarkers. Sci Rep.

[30] WHO (2022).

