# Supplementary material for: Diagnostic accuracy of a three-gene Mycobacterium tuberculosis host response cartridge using fingerstick blood for childhood tuberculosis: a multicentre prospective study in low-income and middle-income countries
Source: Lancet Infect Dis. 2024 Feb;24(2):140–9. doi: 10.1016/S1473-3099(23)00491-7 (PMC10808504; doi:10.1016/S1473-3099(23)00491-7)
Supplement: Supplementary appendix [file mmc1.pdf]

THE LANCET
Infectious Diseases

Supplementary appendix

This appendix formed part of the original submission and has been peer reviewed. We post it as supplied by the authors.

Supplement to: Olbrich L, Verghese VP, Franckling-Smith Z, et al. Diagnostic accuracy of a three-gene *Mycobacterium tuberculosis* host response cartridge using fingerstick blood for childhood tuberculosis: a multicentre prospective study in low-income and middle-income countries. *Lancet Infect Dis* 2023; published online Oct 30. https://doi.org/10.1016/S1473-3099(23)00491-7.

Supplemental Material

"Diagnostic accuracy of a three-gene Mycobacterium tuberculosis host response cartridge using fingerstick blood for childhood tuberculosis: a multicentre prospective study in low-income and middle-income countries"

Contents

Supplemental methods 2

Supplemental Table 1: Inclusion and exclusion criteria for RaPaed-TB study 2

Supplemental Figure 1: Clinical case definitions and reference standards applied in RaPaed-TB................3

Additional definitions 3

Supplemental figures and tables 4

Supplemental Figure 2: Assumption tested for one-way repeated ANOVA for MTB-HR TB score distribution over time. 4

Supplemental Figure 3: Difference in diagnostic accuracy of the MTB-HR TB score in prospective (A) and biobanked (B) by reference standard. 5

Supplemental Figure 4: MTB-HR Ct-distribution of all participants in participants classified by Strict Reference Standard (A), Microbiological Reference Standard (MRS) (B), and Composite Reference Standard (CRS) (C). 6

Supplementary Table 2: Diagnostic accuracy of the MTB-HR TB score cut-off (1.5) for additional RaPaed-TB cohort subgroups against SRS. 7

Supplemental Figure 5: MTB-HR TB score to estimated diagnostic accuracy of MTB-HR in subgroups of interest according to microbiological reference standard (A) and composite reference standard (B).........8

Supplemental Table 3: Diagnostic accuracy of the MTB-HR TB score cut-off (1·5) for the RaPaed-TB cohort subgroups (age, HIV status, malnutrition, TB severity, TB disease location) against MRS. 9

Supplemental Table 4: Diagnostic accuracy of the MTB-HR TB score cut-off (1·5) for the RaPaed-TB cohort subgroups (age, HIV status, malnutrition, TB severity, TB disease location) against CRS. 10

Supplemental Figure 6: Summary ROC curve (SROC) for pooled random-effect meta-analysis accounting for site heterogeneity..11

Supplemental Figure 7: Distribution of MTB-HR TB scores by culture time to positivity and Ultra semi-quantitative result of reference specimen. 11

Supplemental Figure 8: Yield of strategy combining MTB-HR with one or more Ultra tests, among children positive in any respiratory sample (sputum or gastric lavage). 12

Supplemental Figure 9: MTB-HR TB score over time in children with confirmed TB started on TB treatment (A) and children with unlikely TB (B). 12

Supplemental Table 6: MTB-HR as a treatment response marker: clinical characteristics of children who converted from TB positive (MTB-HR TB score < 1·5) to non-TB (MTB-HR TB score ≥ 1·5) at each time point. 13

Supplemental methods

Supplemental Table 1: Inclusion and exclusion criteria for RaPaed-TB study [1]

Abbreviations: CNS = Central nervous system, CSF = Cerebrospinal fluid, IGRA = Interferon-gamma release assay, TST = Tuberculin skin test

Inclusion criteria	Exclusion criteria
1) **<u>Consent and Assent (if applicable):</u>** signed written consent/assent, or witnessed oral consent/assent in the case of illiteracy, before undertaking any study-specific activity.<br>**Of the following, either criterion 2), OR criterion 3), or both, has to be met:**<br>2) **<u>Confirmation of TB disease:</u>** microbiological confirmation of TB disease by positive smear AND/OR culture AND/OR PCR (e.g., Xpert MTB/RIF®); e.g. in a non-study health facility<br>**<u>AND/OR</u>**<br>3) **<u>Signs and Symptoms:</u>** suspicion of TB disease (one or more criteria):<br>a. Chest X-Ray suggestive of TB: <u>cavity</u> AND/OR <u>hilar/mediastinal lymph node</u> enlarged AND/OR miliary pattern<br>b. Weight loss** or failure to thrive within the previous 3 months that, in the investigator's opinion, is not solely due to inadequate feeding; or to another non-TB cause.<br>c. Any cough combined with:<br>• Loss of weight**;<br>• Evidence of *Mycobacterium tuberculosis* infection: TST AND/OR IGRA positive<br>d. Cough alone: persistent unremitting cough duration of ≥ 14 days<br>e. Repeated episodes of fever within 14 days not responding to course of antibiotics AND positive TST or IGRA, (for malaria endemic areas: AND after malaria has been excluded by at least a negative rapid test)<br>f. Signs & symptoms of extrapulmonary TB:<br>• Unilateral non-painful lymph node(s) visibly enlarged ≥ 1 month;<br>• Gibbus (especially of recent onset)<br>• Non-painful enlarged joint<br>• Pleural effusion<br>• Pericardial effusion<br>g. CSF examination findings in line with TB meningitis with at least elevated protein and low glucose (in relation to serum glucose);<br>OR signs and symptoms in line with TB meningitis/CNS TB if lumbar puncture is contraindicated, in the view of the investigator at least one of the following two:<br>• palsy of oculomotoric nerves of recent onset<br>• focal neurological symptoms indicating elevated intracranial pressure OR CNS lesions, of recent onset<br>AND/OR at least two of the following less-specific signs of TB meningitis/CNS TB (for malaria endemic areas: AND a negative malaria rapid diagnostic test*):<br>• Lethargy<br>• Convulsion<br>• Meningism (neck stiffness)<br>• Headache	1) Critical condition (if study procedures seem like an undue risk to the participant's life), such as hypovolemic shock or clinically relevant anaemia (tachypnoea, tachycardia)<br>2) Body weight less than 2 kg<br>3) Children of 15 years of age or more<br>4) Are currently receiving anti-TB drug(s): ideally, eligible participants should not have received any anti-TB treatment.<br>5) In exceptions, up to three daily doses given since treatment start before first study blood draw are acceptable for study inclusion

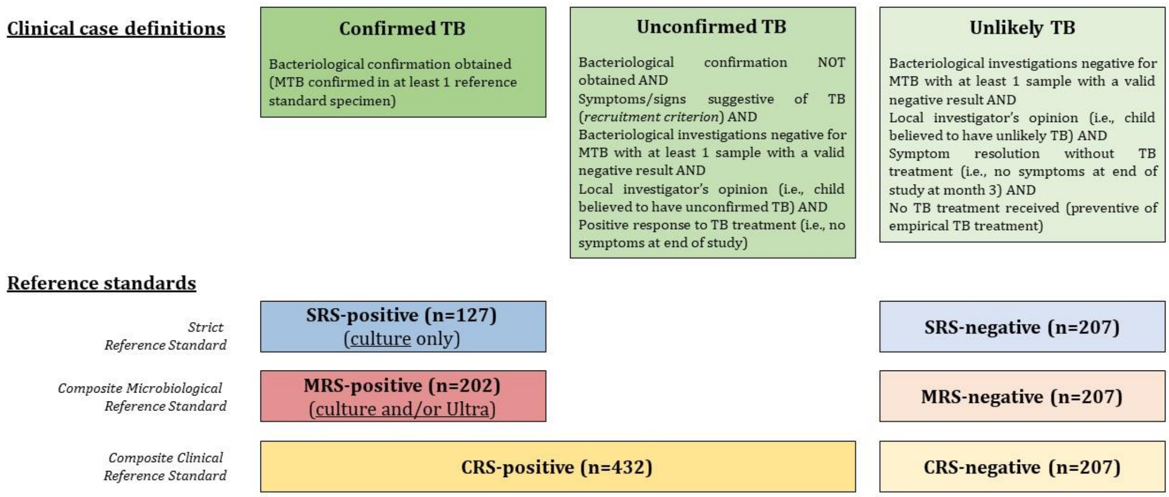

Supplemental Figure 1: Clinical case definitions and reference standards applied in RaPaed-TB
Abbreviations: SRS = Strict Reference Standard , MRS = Composite Microbiological Reference Standard , CRS = Composite Clinical Reference Standard

Additional definitions

Severe/Non-severe TB disease: Definition of non-severe TB disease followed WHO guidelines, being: CXR findings in line with non-severe TB disease (intrathoracic lymph node TB without significant airway obstruction; PTB confined to one lobe with no cavities and no miliary pattern; or uncomplicated pleural effusion); Ultra negative, “Trace”, “Very low” or “Low”; sputum smear negative (if Xpert MTB/RIF or Ultra not available); mild TB symptoms that do not require hospitalization or lethargy. Children living with HIV, with severe acute malnutrition, or under the age of 3 months were excluded.[16]

Supplemental figures and tables

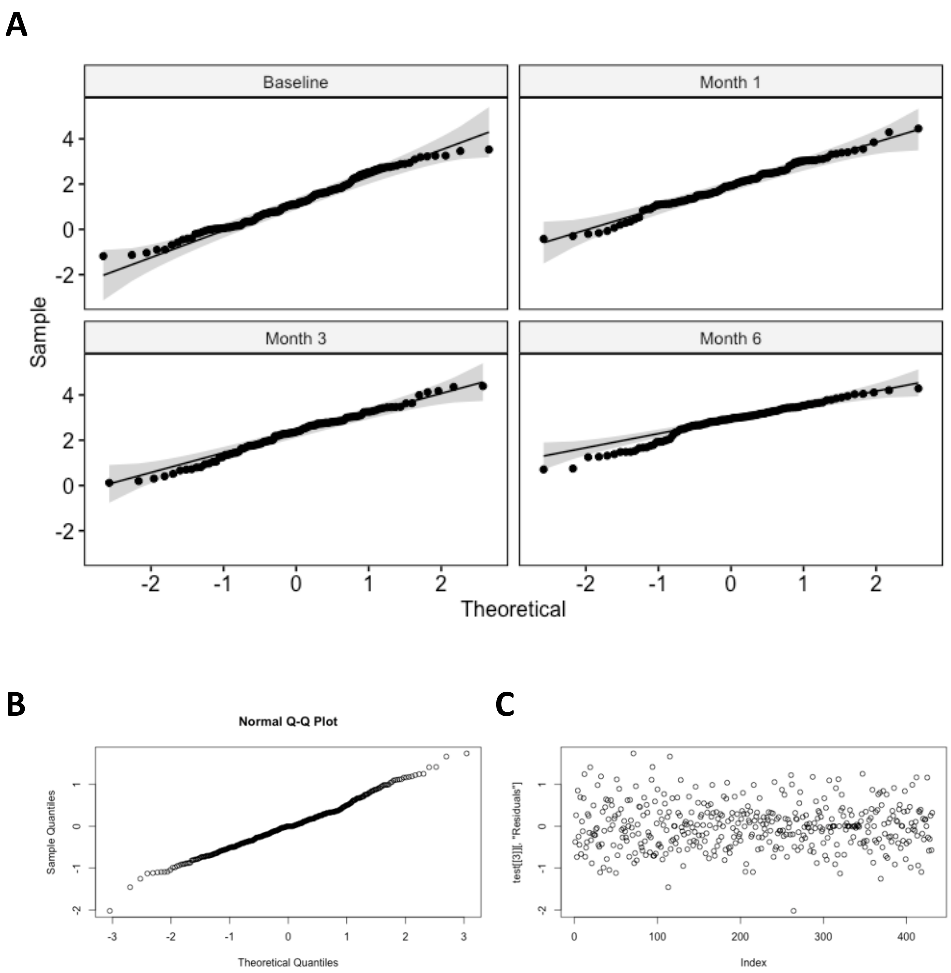

Supplemental Figure 2: Assumption tested for one-way repeated ANOVA for MTB-HR TB score distribution over time.

A. Assumption of normality tested by the Shapiro Wilk test and by plotting residuals. **B** and **C**, assumptions of homoscedasticity tested by plotting the residuals against the predicted values.
The MTB-HR TB score was measured at baseline, month 1, month 3, and at month 6 if the child was on treatment or had persisting symptoms.

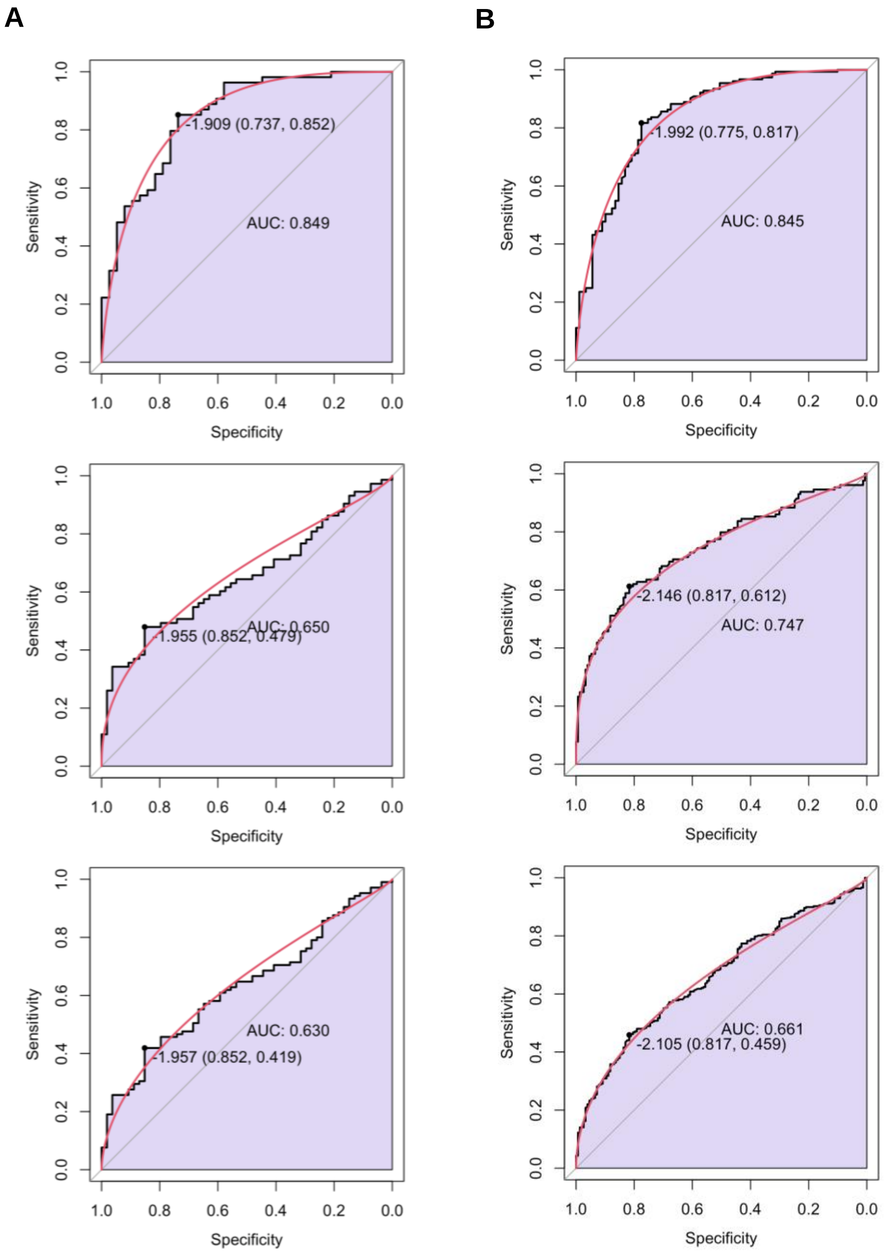

Supplemental Figure 3: Difference in diagnostic accuracy of the MTB-HR TB score in prospective (A) and biobanked (B) by reference standard.

Top. SRS. **Middle.** MRS. **Bottom**. CRS

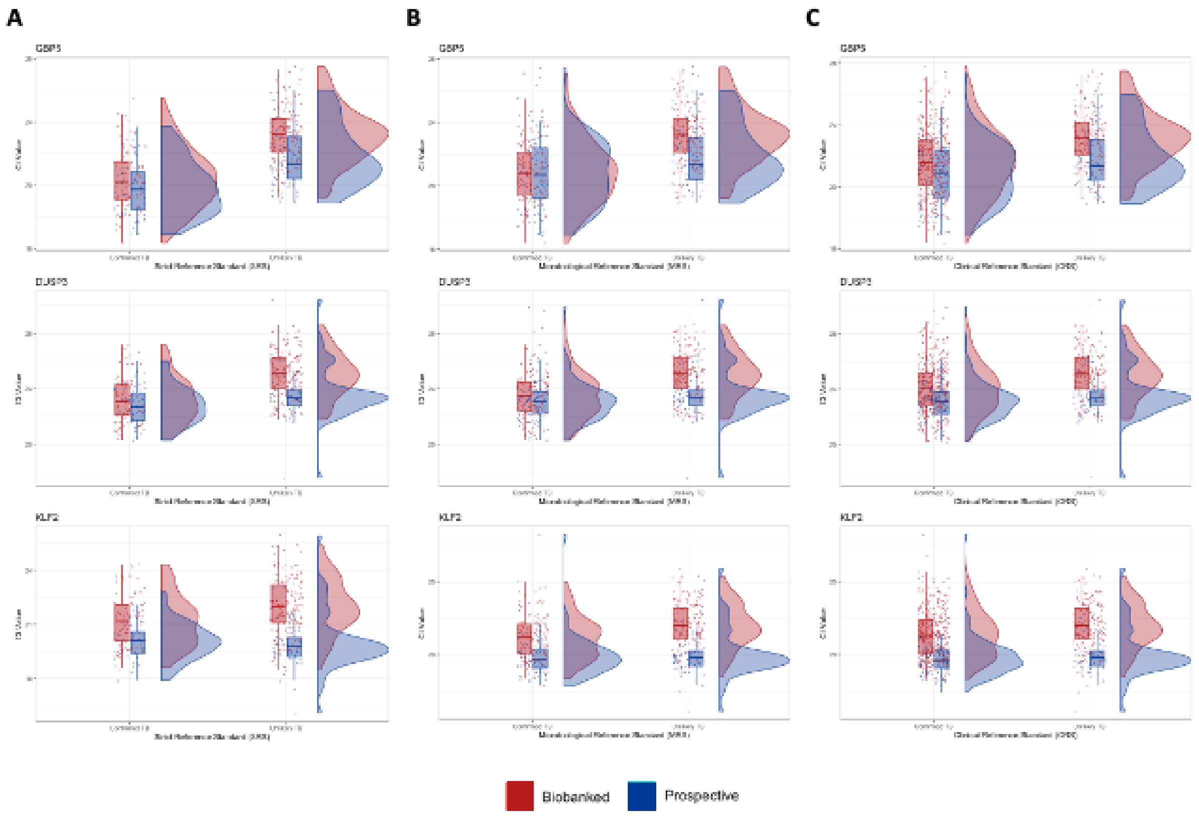

Supplemental Figure 4: MTB-HR Ct-distribution of all participants in participants classified by Strict Reference Standard (A), Microbiological Reference Standard (MRS) (B), and Composite Reference Standard (CRS) (C).

A. SRS. **B.** MRS. **C**. CRS

Raincloud plots show the distribution of the Ct values for the GBP5 (top), DUSP3 (middle), and KLF2 (bottom) gene transcripts, the bars indicate the 10-90% percentile, and the box the interquartile range (IQR). The points are individual data points and show the spread. The data is displayed stratified by whether the sample was prospective (blue) or biobanked (red) (before re-centring).

Supplementary Table 2: Diagnostic accuracy of the MTB-HR TB score cut-off (1.5) for additional RaPaed-TB cohort subgroups against SRS.

The sample size by subgroup is provided (All), number of true positives (TP), false positives (FP), false negatives (FN), and true negatives (TN), along with the sensitivity, specificity, and area under the curve (AUC) with their respective 95% confidence intervals (CIs).

* Denotes missingness

**Sample size was too small to conduct this analysis (< 50, n=4) and (50 - < 200, n=9). Neither had positive results.

Subgroups	All	TP	FP	FN	TN	Sensitivity % (95% CI)	Specificity % (95% CI)
Overall	334	76	20	51	187	59·8 (50·8 – 68·4)	90·3 (85.5 – 94.0)
Sex							
Male	159	33	8	28	90	54·1 (40·8 – 66·9)	91·8 (84·5 – 96·4)
Female	175	43	12	23	97	65·2 (52·4 – 76·5)	89·0 (81·6 – 94·2)
BCG vaccination*							
Not vaccinated	19	8	1	7	3	53·3 (26·6 – 78·7)	75·0 (19·4 – 99·4)
Vaccinated	290	56	18	41	175	57·7 (47·3 – 67·6)	90·7 (85·7 – 94·4)
TB contact in the last months*							
No	143	37	8	28	70	56·9 (44·0 – 69·2)	89·7 (80·8 – 95·5)
Yes	179	37	9	23	110	61·7 (48·2 – 73·9)	92·4 (86·1 – 96·5)
Child ever diagnosed with TB*							
No	323	72	20	50	181	59·0 (49·7 – 67·8)	90·0 (85·1 – 93·8)
Yes	8	4	0	1	3	80·0 (28·4 – 99·5)	100·0 (29·2 – 100·0)
Final TST/Mantoux results*							
Negative	140	14	7	15	104	48·3 (29·4 – 67·5)	93·7 (87·4 – 97·4)
Positive	164	50	10	33	71	60·2 (48·9 – 70·8)	87·7 (78·5 – 93·9)
Hospitalised at enrolment							
No	226	34	14	29	149	54·0 (40·9 - 66·6)	91·4 (86·0 - 95·2)
Yes	108	42	6	22	38	65·6 (52·7 – 77·1)	86·4 (72·6 - 94·8)
Baseline CD4 count							
< 50**	-	-	-	-	-		
50 - < 200**	-	-	-	-	-		
200 - < 500	8	3	2	0	3	100·0 (29·2 – 100·0)	60·0 (14·7 – 94·7)
≥ 500	9	1	0	1	7	50·0 (1·26 – 98·7)	100·0 (59·0 – 100·0)
CXR findings*							
Normal	132	7	8	20	97	25·9 (11·1 – 46·3)	92·4 (85·5 – 96·7)
Abnormal – likely TB	103	53	6	21	23	71·6 (59·9 – 81·5)	79·3 (60·3 – 92·0)
Abnormal equivocal	62	12	5	6	39	66·7 (41·0 – 86·7)	88·6 (75·4 – 96·2)

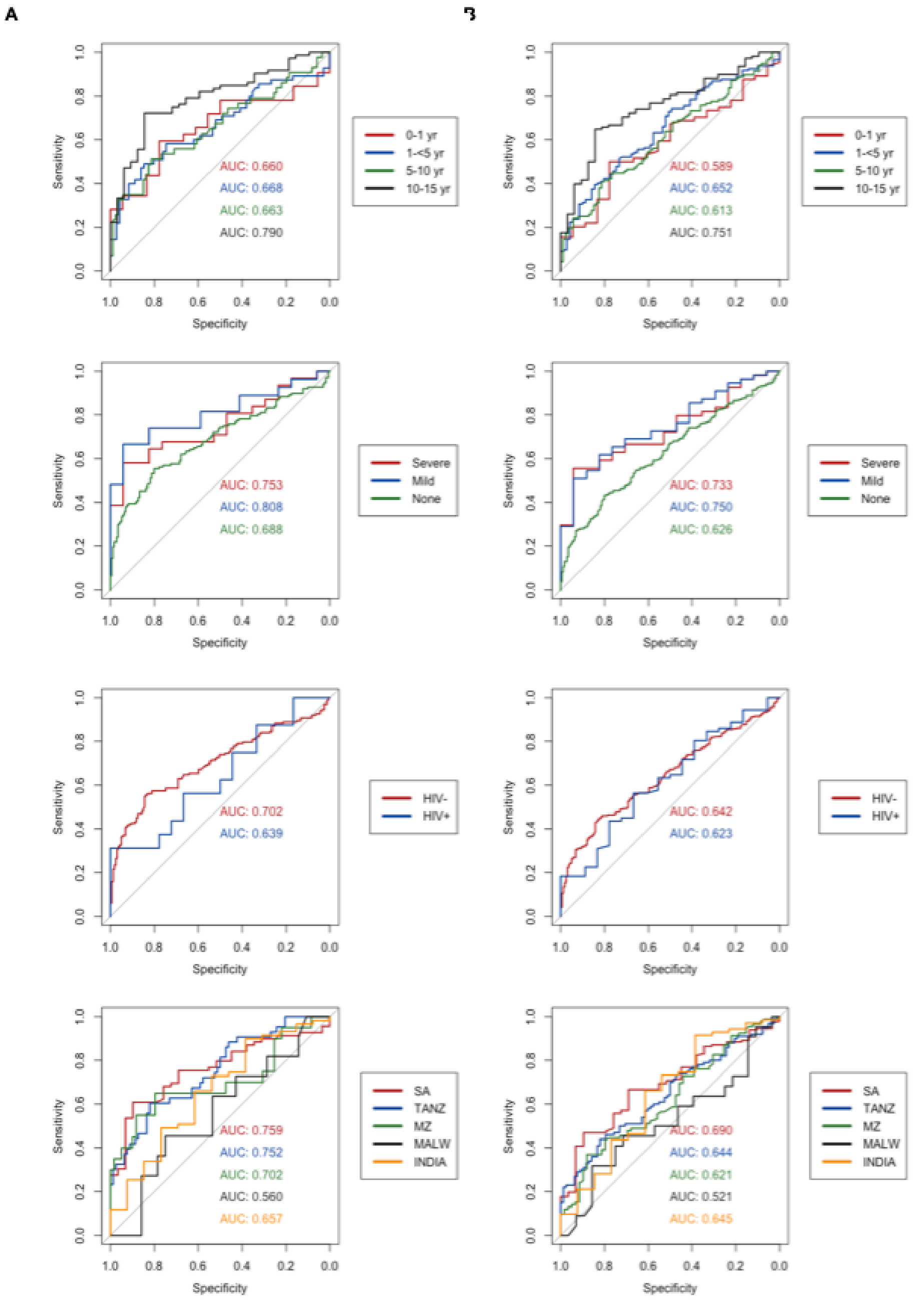

Supplemental Figure 5: MTB-HR TB score to estimated diagnostic accuracy of MTB-HR in subgroups of interest according to microbiological reference standard (A) and composite reference standard (B).

Abbreviations**.** SA: South Africa, TANZ: Tanzania, MZ: Mozambique, MALW: Malawi

A: Age (n=409, n (< 1 year) = 50, n (1-5 years) = 126, n (5-10 years) = 129, n (> 10 years) = 104, DeLong test between Age < 1 and Age 1-5 years: p = 0.932, Age < 1 and Age 5-10 years: p = 0.975, Age < 1 and Age > 10 years: p = 0.151; HIV (N=402, n (negative) = 368, n (positive) = 34, DeLong test between HIV negative and HIV positive: p = 0.542; Malnutrition (N=409, n (none) = 317, n (mild/moderate) = 44, n (severe) = 48, DeLong test between no malnutrition and severe acute malnutrition: p = 0.394 and between no malnutrition and mild/moderate malnutrition: p = 0.566; Site (N=409), n (South Africa) = 98, n (Tanzania) = 121, n (Mozambique) = 79, n (Malawi) = 39, n (India) = 72, DeLong test between South Africa and Tanzania: p = 0.909, South Africa and Mozambique: p = 0.540, South Africa and Malawi: p = 0.089, South Africa and India: p = 0.312.

B: Age (n=639, n (< 1 year) = 82, n (1-5 years) = 215, n (5-10 years) = 202, n (> 10 years) = 140, DeLong test between Age < 1 and Age 1-5 years: p = 0.435, Age < 1 and Age 5-10 years: p = 0.770, Age < 1 and Age > 10 years: p = 0.055; HIV (N=630, n (negative) = 541, n (positive) = 89, DeLong test between HIV negative and HIV positive: p = 0.801; Malnutrition (N=639, n (none) = 496, n (mild/moderate) = 72, n (severe) = 71, DeLong test between no malnutrition and severe acute malnutrition: p = 0.112 and between no malnutrition and mild/moderate malnutrition: p = 0.849; Site (N=639), n (South Africa) = 176, n (Tanzania) = 178, n (Mozambique) = 151, n (Malawi) = 50, n (India) = 84, DeLong test between South Africa and Tanzania: p = 0.462, South Africa and Mozambique: p = 0.297, South Africa and Malawi: p = 0.090, South Africa and India: p = 0.659.

Supplemental Table 3: Diagnostic accuracy of the MTB-HR TB score cut-off (1·5) for the RaPaed-TB cohort subgroups (age, HIV status, malnutrition, TB severity, TB disease location) against MRS.

The sample size by subgroup is provided (All), number of true positives (TP), false positives (FP), false negatives (FN), and true negatives (TN), along with the sensitivity and specificity with their respective 95% confidence intervals (CIs).

* Denotes missingness

** TB disease severity and location imply that the child already has tuberculosis, hence calculating a specificity was not possible

*** The sample size for CD4 category (< 50) was too small and there were no positives.

Subgroups	All	TP	FP	FN	TN	Sensitivity % (95% CI)	Specificity % (95% CI)
Overall	409	84	20	118	187	41·6 (34·7 – 48·7)	90·3 (85·5 – 94·0)
Age							
< 1	50	9	1	23	17	28·1 (13·7 – 46·7)	94·4 (72·7 – 99·9)
1 - 5	126	20	6	35	65	36·4 (23·8 – 50·4)	91·5 (82·5 – 96·8)
5 – 10	129	15	9	28	77	34·9 (21·0 – 50·9)	89·5 (81·1 – 95·1)
> 10	104	40	4	32	28	55·6 (43·4 – 67·3)	87·5 (71·0 – 96·5)
HIV status							
Negative	368	79	17	105	167	42·9 (35·7 – 50·4)	90·8 (85·6 – 94·5)
Positive	34	5	2	11	16	31·3 (11·0 – 58·7)	88·9 (65·3 – 98·6)
Malnutrition							
None	317	55	18	89	155	38·2 (30·2 – 46·7)	89·6 (84·1 – 93·7)
Mild/moderate	44	16	1	11	16	59·3 (38·8 – 77·6)	94·1 (71·3 – 99·9)
Severe	48	13	1	18	16	41·9 (24·5 – 60·9)	94·1 (71·3 – 99·9)
Site							
South Africa	98	38	3	31	26	55·1 (42·6 – 67·1)	89·7 (72·6 – 97·8)
Tanzania	121	17	6	26	72	39·5 (25·0 – 55·6)	92·3 (84·0 – 97·1)
Mozambique	79	8	5	12	54	40·0 (19·1 – 63·9)	91·5 (81·3 – 97·2)
Malawi	39	1	4	10	24	9·1 (0·2 – 41·3)	85·7 (67·3 – 96·0)
India	72	20	2	39	11	33·9 (22·1 – 47·4)	84·6 (54·6 – 98·1)
TB severity**							
Non severe	41	13	-	28	-	31·7 (18·1 – 48·1)	-
Severe	155	70	-	85	-	45·2 (37·2 – 53·3)	-
TB location**							
PTB	111	42	-	69	-	37·8 (28·8 – 47·5)	-
EPTB	39	10	-	29	-	25·6 (13·0 – 42·1)	-
PTB + EPTB	46	31	-	15	-	67·4 (52·0 – 80·5)	-
Lymph node TB	28	15	-	13	-	53·6 (33·9 – 72·5)	-
TB meningitis	20	12	-	8	-	60·0 (36·1 – 80·9)	-
Sex							
Male	204	37	8	69	90	34·9 (25·9 – 44·8)	91·8 (84·5 – 96·4)
Female	205	47	12	49	97	49·0 (38·6 – 59·4)	89·0 (81·6 – 94·2)
BCG vaccination							
Not vaccinated	19	8	1	7	3	53·3 (26·6 – 78·7)	75·0 (19·4 – 99·4)
Vaccinated	359	62	18	104	175	37·3 (30·0 – 45·2)	90·7 (85·7 – 94·4)
TB contact in last months							
No	191	42	8	71	70	37·2 (28·3 – 46·8)	89·7 (80·8 – 95·5)
Yes	205	40	9	46	110	46·5 (35·7 – 57·6)	92·4 (86·1 – 96·5)
Child ever diagnosed w/ TB							
No	396	79	20	116	181	40·5 (33·6 – 47·8)	90·0 (85·1 – 93·8)
Yes	10	5	0	2	3	71·4 (29·0 – 96·3)	100·0 (29·2 – 100·0)
Final TST/Mantoux results							
Negative	180	16	7	53	104	23·2 (13·9 – 34·9)	93·7 (87·4 – 97·4)
Positive	194	56	10	57	71	49·6 (40·0 – 59·1)	87·7 (78·5 – 93·9)
Hospitalised at enrolment							
No	260	38	14	59	149	39·2 (29·4 – 49·6)	91·4 (86·0 – 95·2)
Yes	149	46	6	59	38	43·8 (34·1 – 53·8)	86·4 (72·6 – 94·8)
Baseline CD4 count							
< 50***	-	-	-	-	-		
50 - < 200	4	1	0	1	2	50·0 (1·3 – 98·7)	100·0 (15·8 – 100·0)
200 - < 500	11	3	2	3	3	50·0 (11·8 – 88·2)	60·0 (14·7 – 94·7)
≥ 500	11	1	0	3	7	25·0 (0·6 – 80·6)	100·0 (59·0 – 100·0)
CXR findings							
Normal	126	12	10	37	67	24·5 (13·3 – 38·9)	87·0 (77·4 – 93·6)
Abnormal, likely TB	207	66	8	63	70	51·2 (42·2 – 60·1)	89·7 (80·8 – 95·5)
Abnormal equivocal	50	4	2	13	31	23·5 (6·8 – 49·9)	93·9 (79·8 – 99·3)

Supplemental Table 4: Diagnostic accuracy of the MTB-HR TB score cut-off (1·5) for the RaPaed-TB cohort subgroups (age, HIV status, malnutrition, TB severity, TB disease location) against CRS.

The sample size by subgroup is provided (All), number of true positives (TP), false positives (FP), false negatives (FN), and true negatives (TN), along with the sensitivity and specificity with their respective 95% confidence intervals (Cis).

* Denotes missingness

** TB disease severity and location imply that the child already has tuberculosis, hence calculating a specificity was not possible

*** The sample size for CD4 category (< 50) was too small and there were no positives.

Subgroups	All	TP	FP	FN	TN	Sensitivity % (95% CI)	Specificity % (95% CI)
Overall	639	128	20	304	187	29·6 (25·4 – 34·2)	90·3 (85·5 – 94·0)
Age							
< 1	82	10	1	54	17	15·6 (7·8 – 26·9)	94·4 (72·7 – 99·9)
1 - 5	215	37	6	107	65	25·7 (18·8 – 33·6)	91·5 (82·5 – 96·8)
5 – 10	202	29	9	87	77	25·0 (17·4 – 33·9)	89·5 (81·1 – 95·1)
> 10	140	52	4	56	28	48·1 (38·4 – 58·0)	87·5 (71·0 – 96·5)
HIV status							
Negative	541	115	17	242	167	32·2 (27·4 – 37·3)	90·8 (85·6 – 94·5)
Positive	89	13	2	58	16	18·3 (10·1 – 29·3)	88·9 (65·3 – 98·6)
Malnutrition							
None	496	89	18	234	155	27·6 (22·8 – 32·8)	89·6 (84·1 – 93·7)
Mild/moderate	72	21	1	34	16	38·2 (25·4 – 52·3)	94·1 (71·3 – 99·9)
Severe	71	18	1	36	16	33·3 (21·1 – 47·5)	94·1 (71·3 – 99·9)
Site							
South Africa	176	63	3	84	26	42·9 (34·7 – 51·3)	89·7 (72·6 – 97·8)
Tanzania	178	28	6	72	72	28·0 (19·5 – 37·9)	92·3 (84·0 – 97·1)
Mozambique	151	14	5	78	54	15·2 (8·6 – 24·2)	91·5 (81·3 – 97·2)
Malawi	50	3	4	19	24	13·6 (2·9 – 34·9)	85·7 (67·3 – 96·0)
India	84	20	2	51	11	28·2 (18·1 – 40·1)	84·6 (54·6 – 98·1)
TB severity**							
Non severe	126	26	-	100	-	20·6 (13·9 – 28·8)	-
Severe	292	101	-	191	-	34·6 (29·1 – 40·4)	-
TB location**							
PTB	285	67	-	218	-	23·5 (18·7 – 28·9)	-
EPTB	59	15	-	44	-	25·4 (15·0 – 38·4)	-
PTB + EPTB	74	45	-	29	-	60·8 (48·8 – 72·0)	-
Lymph node TB	46	19	-	27	-	41·3 (27·0 – 56·8)	-
TB meningitis	25	13	-	12	-	52·0 (31·3 – 72·2)	-
Sex							
Male	333	61	8	174	90	26·0 (20·5 – 32·1)	91·8 (84·5 – 96·4)
Female	306	67	12	130	97	34·0 (27·4 – 41·1)	89·0 (81·6 – 94·2)
BCG vaccination							
Not vaccinated	25	9	1	12	3	42·9 (21·8 – 66·0)	75·0 (19·4 – 99·4)
Vaccinated	549	98	18	258	175	27·5 (23·0 – 32·5)	90·7 (85·7 – 94·4)
TB contact in last months							
No	295	64	8	153	70	29·5 (23·5 – 36·0)	89·7 (80·8 – 95·5)
Yes	322	59	9	144	110	29·1 (22·9 – 35·8)	92·4 (86·1 – 96·5)
Child ever diagnosed w/ TB							
No	614	123	20	290	181	29·8 (25·4 – 34·4)	90·0 (85·1 – 93·8)
Yes	22	5	0	14	3	26·3 (9·2 – 51·2)	100·0 (29·2 – 100·0)
Final TST/Mantoux results							
Negative	274	25	7	138	104	15·3 (10·2 – 21·8)	93·7 (87·4 – 97·4)
Positive	299	83	10	135	71	38·1 (31·6 – 44·9)	87·7 (78·5 – 93·9)
Hospitalised at enrolment							
No	414	60	14	191	149	23·9 (18·8 – 29·7)	91·4 (86·0 – 95·2)
Yes	225	68	6	113	38	37·6 (30·5 – 45·1)	86·4 (72·6 – 94·8)
Baseline CD4 count							
< 50***	-	-	-	-	-		
50 - < 200	9	3	0	4	2	42·9 (9·9 – 81·6)	100·0 (15·8 – 100·0)
200 - < 500	30	8	2	17	3	32·0 (14·9 – 53·5)	60·0 (14·7 – 94·7)
≥ 500	32	1	0	24	7	4·0 (0·1 – 20·4)	100·0 (59·0 – 100·0)
CXR findings							
Normal	188	22	10	89	67	19·8 (12·9 – 28·5)	87·0 (77·4 – 93·6)
Abnormal, likely TB	337	97	8	162	70	37·5 (31·5 – 42·7)	89·7 (80·8 – 95·5)
Abnormal equivocal	68	5	2	30	31	14·3 (4·8 – 30·3)	93·9 (79·8 – 99·3)

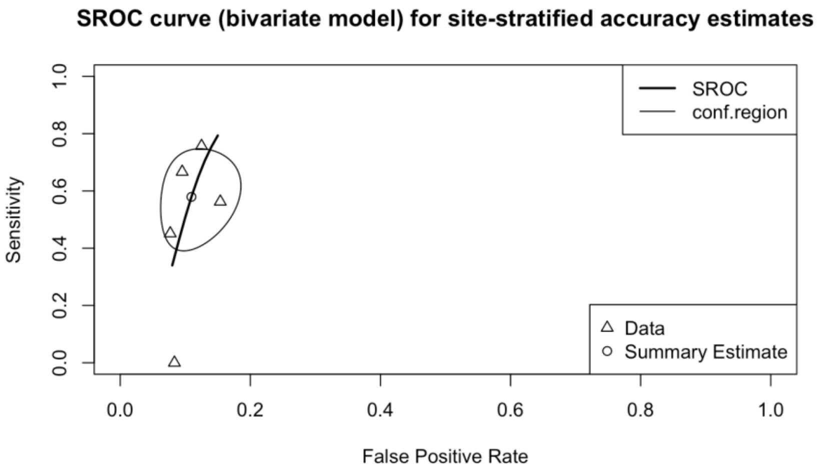

Supplemental Figure 6: Summary ROC curve (SROC) for pooled random-effect meta-analysis accounting for site heterogeneity

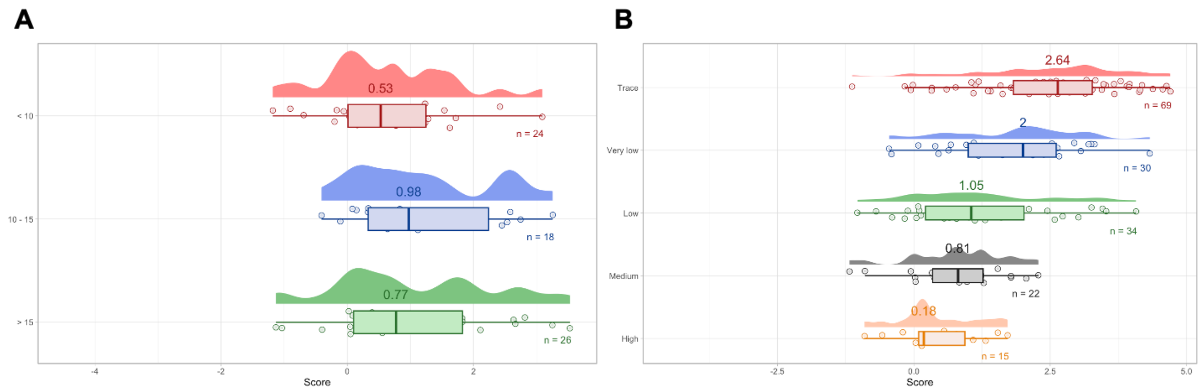

Supplemental Figure 7: Distribution of MTB-HR TB scores by culture time to positivity and Ultra semi-quantitative result of reference specimen.

A. Distribution of MTB-HR TB score across groups of MGIT time to positivity (< 10 days, 10-15 days, and > 15 days) among those that had a positive culture. Raincloud plots show the distribution, and the bars indicate the 10-90% percentile, the box the interquartile range (IQR). The points are individual data points and show the spread. **B**. Distribution of MTB-HR TB score by Ultra semi-quantitative result of sputum sample (spontaneous or induced). Of 171 children with microbiologically confirmed TB, 84 (48·9%) were confirmed by both Ultra and TB culture, 62 (36·0%) by Ultra only, and 26 (15·1%) by TB culture only. For those 84 children confirmed by both Ultra and TB culture, the semi-quantitative Ultra readout "Trace" was observed in 12 (14·3%), "Very low" in 16 (19·1%), "Low" in 27 (32·1%), "Medium" in 16 (19·0%), and "High" in 12 (14·3%), with one semiquantitative readout missing. In contrast, observed strength of signal in 62 children confirmed by Ultra alone was lower - mostly "Trace" in 46 (74·2%), "Very low" in 8 (12·9%), "Low" in 6 (9·7%), and "Medium" in 2 (3·2%). Notably, of those 62 children only confirmed by "Trace", 60 (96·8%) were only confirmed by one single "Trace" call. Raincloud plots show the distribution, and the bars indicate the 10-90% percentile, the box the interquartile range (IQR). The points are individual data points and show the spread.

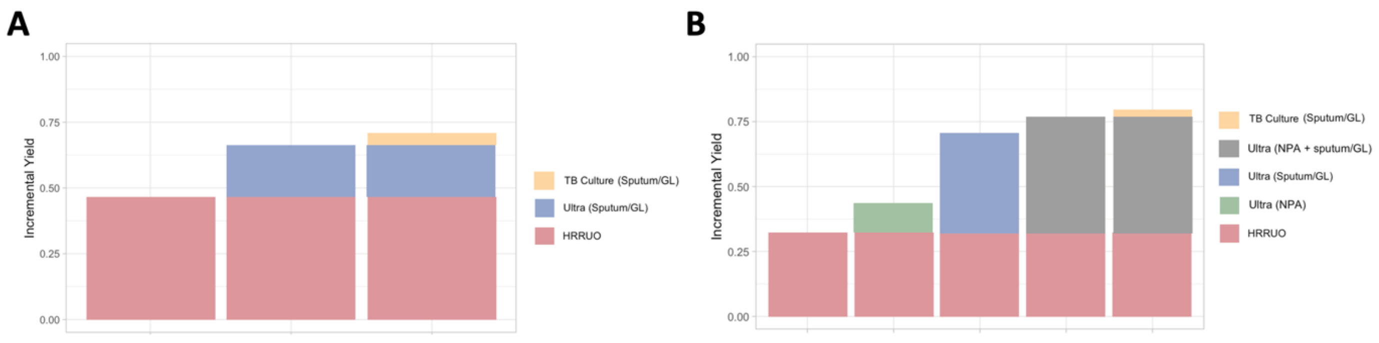

Supplemental Figure 8: Yield of strategy combining MTB-HR with one or more Ultra tests, among children positive in any respiratory sample (sputum or gastric lavage).

A. Children 5-14 years: incremental yield of adding one sputum Ultra, and a subsequent sputum culture(n=109). In older children 5-14 years, 50/109 (45·9%) were identified by a positive MTB-HR, with an additional 20/109 (18·3%) testing positive on sputum/GL Ultra. TB culture identified only 5/109 (4·6%) in addition

B. Children <5 years: incremental yield of adding one Ultra on NPA, followed by one Ultra and a subsequent culture on sputum and/or gastric lavage for children < 5 years if microbiologically confirmed (n=60). Among young children (<5 years), 19/60 (31·7%) tested positive by MTB-HR, with an additional 3/60 (5·0%) identified by one positive Ultra on NPA alone, 20/60 (33·3%) by a positive Ultra on a respiratory sample (sputum/GL) alone, and 27/60 (45·0%) by a positive Ultra on a respiratory sample and/or NPA. The additional yield of a TB culture in addition to Ultra was low (n=1, 1·7%).

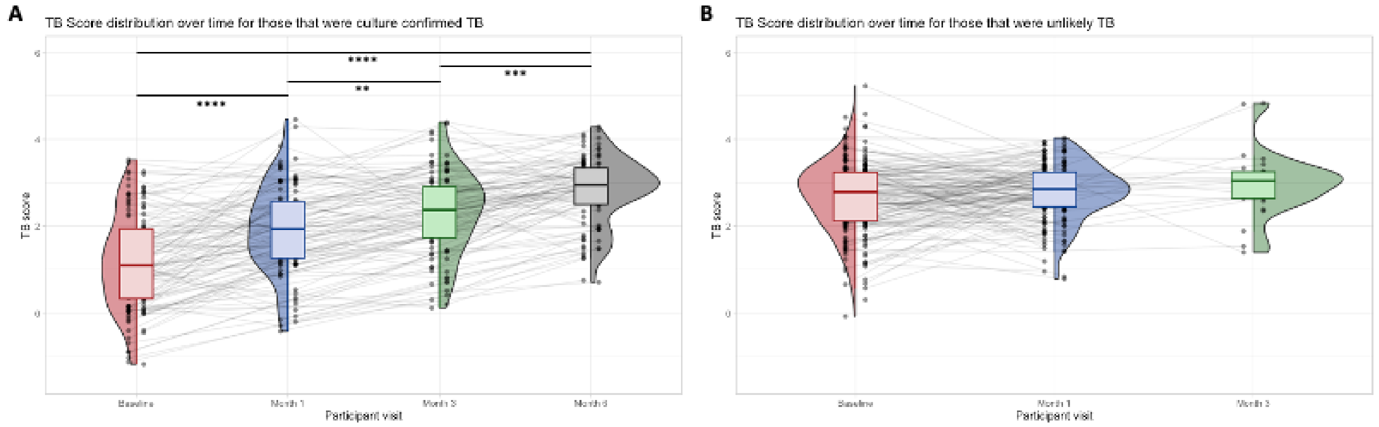

Supplemental Figure 9: MTB-HR TB score over time in children with confirmed TB started on TB treatment (A) and children with unlikely TB (B).

Rain Cloud plots show the distribution and the box the interquartile range (IQR) along with the median. The points are individual data points and connected over time showing the trends. Bars with asterisks (*) represent the results of the pairwise paired t-tests conducted between visits. *p < 0.05 *, p < 0.01 **, p < 0.001 ***, p < 0.0001 ****.*

Supplemental Table 6: MTB-HR as a treatment response marker: clinical characteristics of children who converted from TB positive (MTB-HR TB score < 1·5) to non-TB (MTB-HR TB score ≥ 1·5) at each time point.

Total: children who were TB positive at the baseline visit (n=76). The numbers reflect the numbers of children who converted within the subgroup and time point and the denominator for the proportion is n=76. Those where n of subcategories is missing are missing datapoints and excluded from analysis.

**Subgroups**	n	**All (%)**	**1 month (%)**	**3 months (%)**	**6 months (%)**
All	76	59 (77·6)	32 (42·1)	13 (17·1)	14 (18·4)
Age (categorised)					
< 1	8	7 (87·5)	6 (75·0)	1 (12·5)	0 (0·0)
1 – 5	19	17 (89·5)	9 (47·4)	2 (10·5)	6 (66·7)
5 – 10	14	10 (71·4)	7 (50·0)	2 (14·3)	1 (7·1)
10 – 15	35	25 (71·4)	10 (28·6)	8 (22·9)	7 (20·0)
HIV status					
Negative	62	50 (80·6)	27 (43·5)	12 (19·4)	11 (17·7)
Positive	4	0 (0·0)	0 (0·0)	0 (0·0)	0 (0·0)
Malnutrition					
None	46	37 (80·4)	20 (43·5)	9 (19·6)	8 (17·4)
Mild/moderate	13	11 (84·6)	4 (30·8)	2 (15·4)	5 (38·5)
Severe	12	7 (58·3)	5 (41·7)	1 (8·3)	1 (8·3)
Site					
UCTLI	35	30 (85·7)	17 (48·6)	7 (20·0)	6 (17·1)
MMRC	14	14 (100·0)	10 (71·4)	3 (21·4)	1 (7·1)
INS	7	5 (71·4)	1 (14·3)	1 (14·3)	3 (42·9)
CoM	1	1 (100·0)	0 (0·0)	0 (0·0)	1 (100·0)
CMC	18	9 (50·0)	4 (22·2)	2 (11·1)	3 (16·7)
TB disease severity					
Non severe TB	10	10 (100·0)	8 (80·0)	1 (10·0)	1 (10·0)
Severe TB	66	48 (72·7)	23 (34·8)	12 (18·2)	13 (19·7)

References

1. Olbrich L, Nliwasa M, Sabi I, et al. Rapid and Accurate Diagnosis of Pediatric Tuberculosis Disease (RaPaed-TB): A Diagnostic Accuracy Study for Pediatric Tuberculosis. *The Pediatric Infectious Disease Journal* 2023: e003853.
